# *Encyonopsis indonesica* sp. nov. (Bacillariophyceae, Cymbellales), a new diatom from the ancient lake Matano (Sulawesi, Indonesia)

**DOI:** 10.3897/phytokeys.175.61044

**Published:** 2021-03-17

**Authors:** Dmitry A. Kapustin, Anton M. Glushchenko, John P. Kociolek, Maxim S. Kulikovskiy

**Affiliations:** 1 Timiryazev Institute of Plant Physiology, Russian Academy of Sciences, 127276, Moscow, Russia Timiryazev Institute of Plant Physiology, Russian Academy of Sciences Moscow Russia; 2 Museum of Natural History, Boulder, Colorado, 80309, USA Museum of Natural History Boulder United States of America; 3 Department of Ecology and Evolutionary Biology University of Colorado, Boulder, Colorado, 80309, USA Biology University of Colorado Boulder United States of America

**Keywords:** ancient lake, diatoms, Indonesia, morphology, SEM, taxonomy

## Abstract

A new species, *Encyonopsis indonesica*, is described from the ancient lake Matano, Sulawesi island, Indonesia. The morphology of this species was studied by means of light and scanning electron microscopy. *E. indonesica* has a remarkable valve ultrastructure. The valve surface is ornamented with numerous longitudinal siliceous ribs and siliceous verrucae. Valve face delineated from the mantle by a thickened marginal ridge. Raised sterna border the raphe branches. Raphe is distinctly undulate with distal ends hooked strongly to the ventral side. The only similar species to *E. indonesica* is *Amphora dissimilis* described from New Caledonia. Comparison of both taxa is given and *A. dissimilis* is transferred to *Encyonopsis*. The taxonomic placement of both taxa is evaluated, and the phenomenon of external siliceous ornamentation is discussed.

## Introduction

The genus *Encyonopsis* Krammer was established during a comprehensive revision of cymbelloid diatoms by Krammer (1997a). It differs from morphologically related genus *Encyonema* Kützing mainly by its slightly dorsiventral valve outline and by terminal raphe fissures only slightly bent to the ventral margin (Krammer 1997a, b; Potapova 2014). Though described less than a quarter of a century ago, over 170 taxa have been assigned to *Encyonopsis* (Kociolek et al. 2020). Many taxa were transferred by Krammer (1997b) from other genera in his initial circumscription of the genus, but since then new species of the genus have been described from western North America (Bahls 2013; Graeff and Kociolek 2013; Kociolek et al. 2014; Mark et al. 2019), Central America (Wydrzycka and Lange-Bertalot 2001), South America (Metzeltin and Lange-Bertalot 2007; Wengrat et al. 2015), Europe (Van de Vijver et al. 2012; Kennedy et al. 2019), Asia (Krammer 2003; Potapova et al. 2014), Madagascar (Metzeltin and Lange-Bertalot 2002), and Reunion Island (Le Cohu et al. 2014).

Relatively recently the genus *Kurtkrammeria*Bahls (2015) was proposed, with most species of the genus having been transferred from *Encyonopsis*. This genus differs from *Encyonopsis* by having convergent striae at the apices, slit-like apically-oriented or crescent-shaped areolae, the internal proximal raphe ends hooked strongly towards the dorsal side of the valve and the presence (or absence) of stigmata (Bahls 2015; Marquardt et al. 2016; Zhang et al. 2020). Despite the rather weak differences, *Kurtkrammeria* is an accepted genus and several new species have been described since its description (Marquardt et al. 2016; Zhang et al. 2020). However, sometimes taxa of *Gomphonema* are misidentified as *Encyonopsis* or *Kurtkrammeria* (e.g., Bahls et al. 2018; Almeida et al. 2020), suggesting a review of the distinguishing features of these genera may be warranted.

The Malili lakes are located in the mountains of Central Sulawesi and are composed of five tectonic lakes, namely, Matano, Mahalona, Towuti, Lontoa (also known as Wawontoa) and Masapi (Brooks 1950; Vaillant et al. 2011; von Rintelen et al. 2012). Lake Matano is the oldest lake among them, and its estimated age is 2 to 4 million years old (Brooks 1950; Vaillant et al. 2011). The most extensive treatment on diatoms from the Malili lakes was performed by Hustedt (1942). Subsequently, many new taxa were described from these lakes (Bramburger et al. 2006; Kociolek et al. 2018; Kapustin et al. 2019) and some taxa were re-investigated (Kapustin et al. 2017; Kapustin and Kulikovskiy 2018; Kapustin et al. 2020; Kulikovskiy et al. 2020).

The aim of this paper is to describe a new *Encyonopsis* species based on light and scanning electron microscopy, detail its unusual morphology, and discuss its generic placement.

## Material and methods

An epilithic sample containing *Encyonopsis indonesica* was collected from Lake Matano in 2010 (02°28.433'S, 121°15.710'E). With a Hanna multiparameter probe meter (HANNA HI98128), the temperature was recorded as 28.5 °C, pH as 8.53, and conductivity as 177 μS∙cm^–1^.

The sample was heated in concentrated hydrogen peroxide (~37%) to dissolve the organic matter. It was then rinsed with deionized water four times at 12 h intervals. After decanting and filling with deionized water up to 100 ml, the suspension was spread on to coverslips and left to dry at room temperature. Permanent diatom slides were mounted in Naphrax. Light microscopic (LM) observations were performed with a Zeiss Scope A1 microscope equipped with an oil immersion objective (100×/n.a.1.4, differential interference contrast [DIC]) and Zeiss Axio-Cam ERc 5s camera. Valve ultrastructure was examined by means of a JSM-6510LV scanning electron microscope (Papanin Institute for Biology of Inland Waters RAS, Borok, Russia). For scanning electron microscopy (SEM), parts of the suspensions were fixed on aluminum stubs after air-drying. The stubs were sputter coated with 50 nm of gold.

## Results

### Class Bacillariophyceae Haeckel

#### Order Cymbellales D.G. Mann

**Family Cymbellaceae Kützing**

**Genus *Encyonopsis* Krammer**

##### Encyonopsis indonesica

Taxon classificationPlantaeCymbellalesCymbellaceae

Kapustin, Kulikovskiy & Kociolek sp. nov.

980C107F-B179-5BD3-8CAF-CCC821C99AB4

Figs 1 , 2 , 3 , 4

###### Holotype

**Figure 1.**
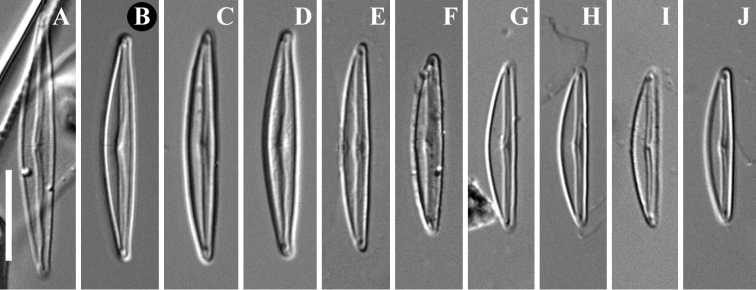
**A–J***Encyonopsis indonesica* sp. nov. (LM). Size diminution series showing variation in valve outline **B** holotype specimen. Scale bar: 10 μm.

(here designated): MHA 01105. Fig. 1B illustrates the holotype.

###### Type locality.

Indonesia, Island of Sulawesi, Lake Matano, 02°28.433'S, 121°15.710'E, *leg.* I.I. Ivanov, 14.XI.2010.

###### Etymology.

The specific epithet refers to the type locality from Indonesia.

###### Description.

**LM (Fig. 1A–J).** Valves dorsi-ventral, semi-lanceolate, with a slightly convex dorsal margin and a nearly straight ventral margin. Apices slightly protracted, cuneate to slightly rostrate. Axial area very narrow, following the course of the raphe and central area not expressed. Raphe undulate. Striae indistinct in LM. Length 17–29 µm (21.9 ± 3.3; n = 16), breadth 3–4 µm (3.4 ± 0.3; n = 16), length/width ratio 5.7–7.4 (6.5 ± 0.6; n = 16).

**Figure 2.**
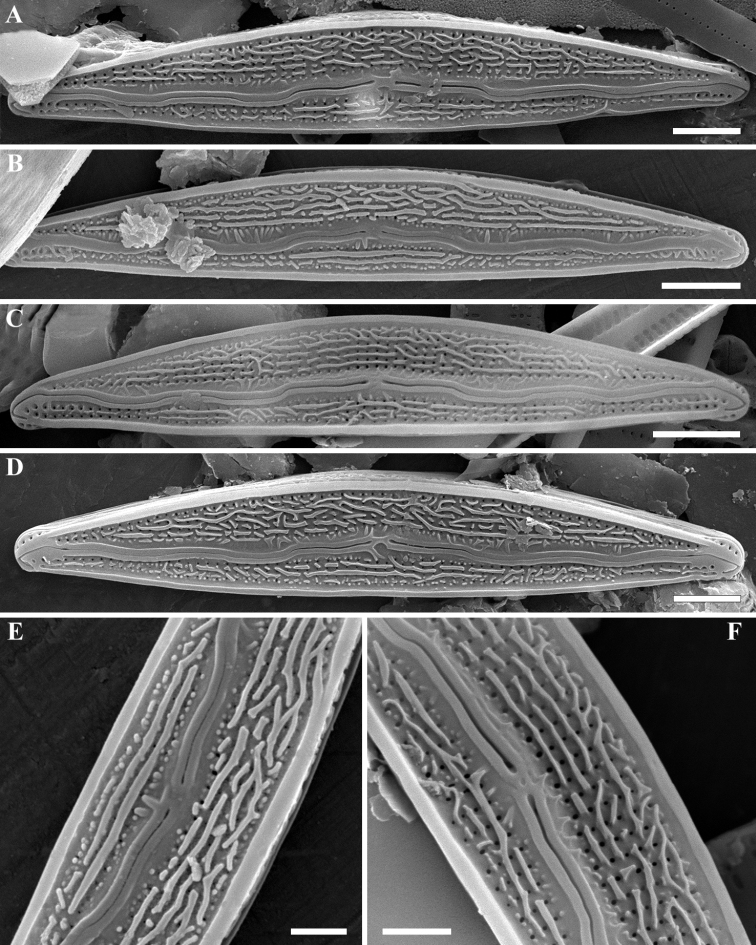
**A–F***Encyonopsis indonesica* sp. nov. (SEM). External view **A–D** whole valves showing morphological variability in external ornamentation **E, F** central area with dorsally deflected proximal raphe ends. Note the thickened marginal ridge, longitudinal ribs and verrucae on the valve surface. Scale bars: 2 µm (**A, D**), 2.5 µm (**B, C**), 1 µm (**E, F**).

**Figure 3.**
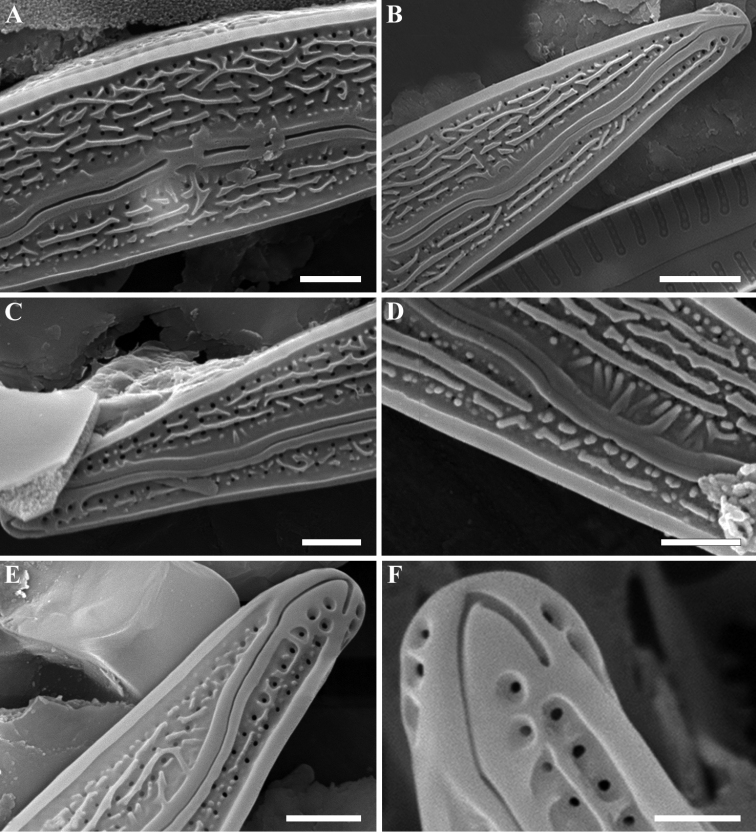
**A–F***Encyonopsis indonesica* sp. nov. (SEM). External view **A** central area **B, C** valve ends. Note a hyaline area along the raised sternum **D** a part of a raphe with transapical ribs on the valve surface **E, F** valve ends with strongly hooked distal raphe fissures. Scale bars: 1 µm (**A, C, D, E**), 2 µm (**B**), 0.5 µm (**F**).

**Figure 4.**
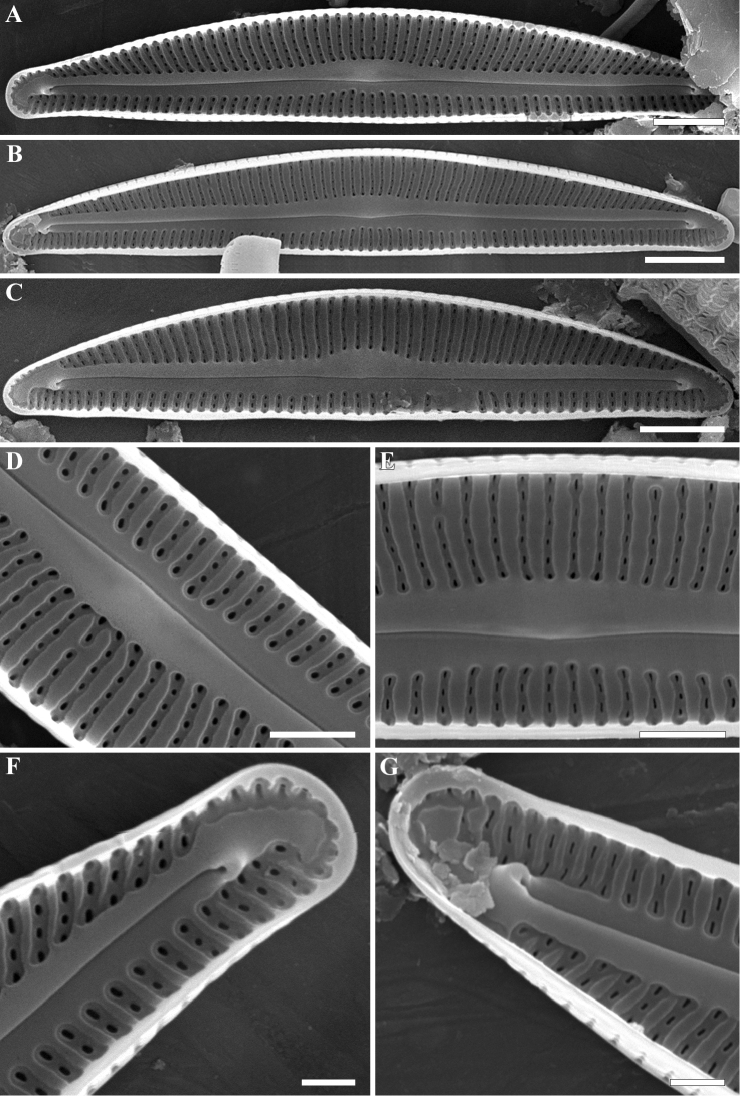
**A–G***Encyonopsis indonesica* sp. nov. (SEM). Internal view **A–C** whole valve **D, E** central area with rounded or elongated areolae located in a transapically elongated grooves **F, G** valve ends with typical helictoglossa. Note a thin, plate-like silica thickening present between helictoglossa and apex. Scale bars: 2 µm (**A, C**), 2.5 µm (**B**), 1 µm (**D, E**), 0.5 µm (**F, G**).

**SEM (Figs 2–4).** External valve face occasionally covered with siliceous verrucae and numerous siliceous ribs of different lengths mostly aligned along the apical axis (Fig. 2A–F). Valve face delineated from the mantle by a thickened marginal ridge. Raphe distinctly undulate, with the proximal raphe ends deflected slightly towards the dorsal margin and the distal ends hooked strongly to the ventral side (Fig. 3E, F). Raised sterna border the raphe branches. Striae almost parallel at the valve center becoming weakly radiate towards the apices, 34–36 in 10 µm. Striae composed of 2–4 areolae on the ventral side and 5–6 areolae on the dorsal side. Areolae rounded and unoccluded, being occasionally slightly smaller near the valve margins, 50 in 10 µm. Internally, areolae rounded or transapically elongated located in a shallow groove. Internal proximal raphe ends obscured; distal raphe ends terminate in well-developed helictoglossae (Fig. 4A–E). A thin, plate-like silica thickening present between helictoglossa and apices (Fig. 4F, G).

## Discussion

*Encyonopsis indonesica* is very similar to *Amphora dissimilis* described from New Caledonia (Moser et al. 1998), however the latter is slightly larger (length 29–40 µm; breadth 4.6–5.4 µm) and has a more arched dorsal margin and more attenuated apices. Additionally, *E. indonesica* is more finely striated and has 34–36 striae in 10 µm whereas *A. dissimilis* has 18–20 striae in 10 µm, which are discernible under LM. External valve structure of both species looks very similar as well. The valve face is delineated from the mantle by a thickened marginal ridge. In *A. dissimilis* the valve face is more heavily silicified, the longitudinal siliceous ribs are longer and thicker (they are even discernible in LM) and they are present in smaller numbers than in *E. indonesica*. In *A. dissimilis* the raphe is almost straight and filiform. It is located close to the ventral side. At the valve center the raphe branches are abruptly curved dorsally to form an arc. Whereas in *E. indonesica* the raphe is distinctly undulate and does not form an arc at the valve center. Unfortunately, the internal valve structure of *A. dissimilis* remains unknown. Morphological and morphometric features of both species are summarized in Table 1.

**Table 1.** Morphological and morphometric comparisons among *Encyonopsis indonesica* and *E. dissimilis*.

Taxon	Valve shape	Valve / ends	Raphe	Valve length, µm	Valve width, µm	Striae in 10 µm	Areolae in 10 µm
*Encyonopsis indonesica*	semi-lanceolate	cuneate to rostrate	undulate	17–29	3–4	34–36	50
*Encyonopsis dissimilis*	semi-lanceolate	cuneate	filiform, proximal ends are abruptly curved dorsally to form an arc at the valve center	29–40	4.6–5.4	18–20	no data

The authors initially had some doubts when placing *Amphora dissimilis* in *Amphora* (Moser et al. 1998, pp. 90, 91). Levkov (2009) included this species in his monograph on *Amphora* but placed it with other species with uncertain systematic positions. He pointed out that the raphe system of *Amphora dissimilis* significantly differs from that of *Amphora* sensu stricto, however, he decided to keep this species in *Amphora* pending additional observations (Levkov 2009).

We agree that *Amphora dissimilis* does not belong to this genus. Taking into account the remarkably similar morphology between *Encyonopsis indonesica* and *Amphora dissimilis* we propose to transfer the latter species to *Encyonopsis*:

***Encyonopsis dissimilis* (Metzeltin & Krammer) Kapustin, Kulikovskiy & Kociolek, comb. nov.**

Basionym: *Amphora dissimilis* Metzeltin & Krammer in Moser et al. 1998. Biblioth. Diatomol. 38: 90, pl. 43: figs 1–8.

In terms of valve outline, the degree of asymmetry about the apical axis might suggest we assign *Encyonopsis indonesica* and *E. dissimilis* to *Cymbellopsis* Krammer rather than to *Encyonopsis*. All species of the genus *Cymbellopsis* have distinctly dorsiventral valve outlines whereas the species of the genus *Encyonopsis* have only slightly dorsiventral valve outlines. However, in *Cymbellopsis* taxa possess an intermissio of Type 1, similar to what is seen in *Encyonema silesiacum* (Krammer 1997a). *Encyonopsis indonesica*, however, lacks an intermissio. Additionally, in *Cymbellopsis* the areolae are internally occluded with hymens whereas in *Encyonopsis indonesica* areolae are unoccluded. Moreover, internally *Encyonopsis indonesica* possesses typical *Encyonopsis* morphology. All described *Cymbellopsis* taxa are restricted to South America and Africa (Kociolek 2018).

Remarkably, many endemic diatoms from Lake Matano, e.g. *Celebesia distinguenda* (Hustedt) Kapustin, Kulikovskiy & Kociolek, *Cymbella eunotioformis* Kapustin, Kociolek & Kulikovskiy, *C. densigranulata* Kapustin, Kociolek & Kulikovskiy, *Gomphonema matanense* Kapustin, Kociolek & Kulikovskiy and *Encyonopsis indonesica* are additionally ornamented with siliceous outgrowths, ridges, granules, or spines (Kapustin et al. 2017, 2019; Kociolek et al. 2018). This situation was also shown for another cymbelloid taxon, in a species of *Delicatophycus* Wynne (Le Cohu et al. 2020) described from New Caledonia. Since silicification in diatoms is genetically encoded (e.g. Kröger 2007) and the ornamentation is rather stable and apparently does not vary significantly, it is possible this feature can be used for species delimitation.

## Supplementary Material

XML Treatment for Encyonopsis indonesica
